# A Rare Cause of Popliteal Artery and Vein Transection: A Case Report and Literature Review

**DOI:** 10.7759/cureus.49591

**Published:** 2023-11-28

**Authors:** Shamon Gumbs, Maria Guevarra-Kissel, Gonzalo Ausqui, Sharique Nazir, Brian Donaldson

**Affiliations:** 1 General Surgery, Columbia University College of Physicians and Surgeons, Harlem Hospital Center, New York, USA; 2 Minimally Invasive Surgery/General Surgery, NYC Health +Hospitals/Harlem, New York, USA

**Keywords:** popliteal vein, popliteal artery transection, extremity vascular trauma, blunt vascular trauma, popliteal injury, trauma

## Abstract

We present a rare occurrence of popliteal vascular injury due to blunt trauma. The patient had an isolated blunt lower extremity trauma. The patient subsequently experienced moderate tenderness and non-expanding hematoma at the popliteal fossa, reduced range of motion at the knee, and diminished distal pulses. X-rays showed a patella dislocation and tibial plateau non-displaced fracture but no knee dislocation. CT angiography showed an abrupt non-opacification of the distal portion of the popliteal artery with an overlying large hematoma. Surgical exploration was performed which revealed a concomitant transection of the popliteal artery and vein with a 5 cm defect. It was repaired with an interposition graft, and a fasciotomy was also performed. Literature has noted that although the overall incidence of popliteal injuries is low, when present due to blunt trauma there is increased morbidity. A high index of suspicion is recommended for vascular injuries in all patients with blunt trauma to the lower extremities. Minimizing time to diagnosis and intervention for limb salvage and improved outcomes.

## Introduction

Extremity vascular injuries secondary to penetrating or blunt trauma occur in approximately 5% of cases. Blunt trauma to the lower extremity has an approximately 28-46% chance of injury to the popliteal vessel injury (PVI) in the form of intimal injury, occlusion, or transection [1,2]. These injuries occur due to traction or avulsion of the vessel or directly due to bony fragments [3]. Based on the National Trauma Data Bank review by Mullenix, PVI actually had an incidence of 0.2% [4]. Knee dislocations are most often associated with popliteal trauma [5]. Historically, there was a high risk of limb loss with these injuries, as most were managed with ligation, resulting in high amputation rates [2].

The diagnosis of lower extremity vascular injuries can be reliably made by physical examination (evaluating for “hard” or “soft” signs) and confirmed by CT angiography. The presence of "hard" signs of vascular injury has a 92-95% sensitivity for injuries requiring intervention. These "hard" signs include bruit or thrill, active or pulsatile hemorrhage, pulsatile or expanding hematoma, signs of limb ischemia, and diminished or absent pulses. The presence of "soft" signs of vascular injury is less useful at predicting major vascular injuries that require intervention. These "soft signs include hypotension or shock, neurologic deficit, stable, non-pulsatile hematoma, and proximity of the wound to major vascular structures [6].

A high index of suspicion of PVI trauma is warranted for all patients with knee dislocations or fractures (especially proximal tibial fractures). Blunt PVI is associated with increased morbidity compared to penetrating trauma, as they are often difficult to diagnose and can be associated with extensive soft tissue injury and bone destruction [3]. Surgical management has drastically evolved over the decades, ranging from ligation with subsequent amputation to limb salvage with early attempts of vascular repair [3]. Repair of concurrent popliteal venous injuries is controversial. Some authors recommend venous repair, as it may enhance venous drainage and therefore reduce the rates of compartment syndrome and amputation. Whereas other authors have experienced no complications related to popliteal venous ligation [3]. The vein may be ligated in situations of hemodynamic instability, and there is a focus on damage control interventions.

This paper highlights the importance of maintaining a high index of suspicion for the diagnosis and prompt management of PVI, which can present subtly in the context of blunt lower extremity trauma and non-displaced fractures or no dislocations.

## Case presentation

We present a case of a 40-year-old male who was brought in after being struck by a motor vehicle. He reported that mainly his left leg was affected. The patient reported immediate pain in the left lower extremity (LLE) and an inability to walk.

**Figure 1:**
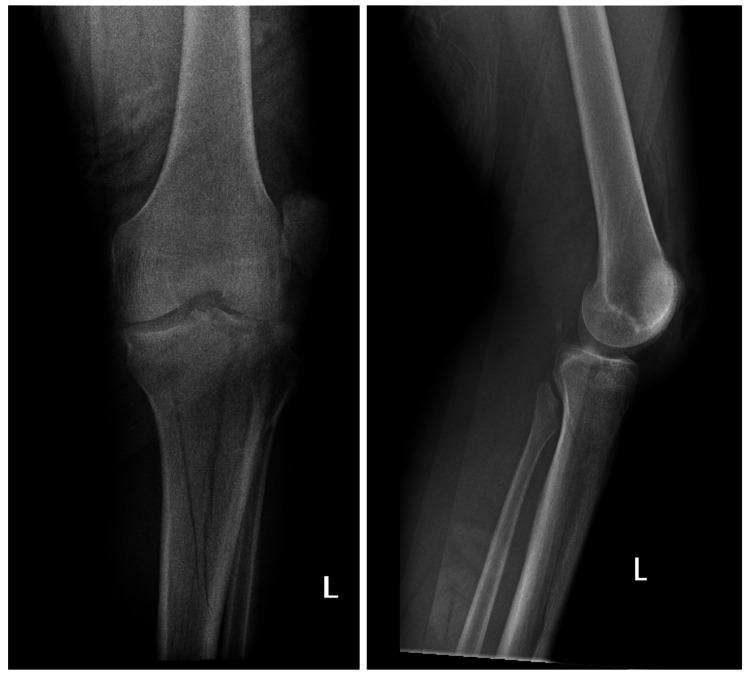
AP and lateral views of the left knee X-ray showing a bicondylar tibial plateau fracture and lateral patella dislocation

**Figure 2:**
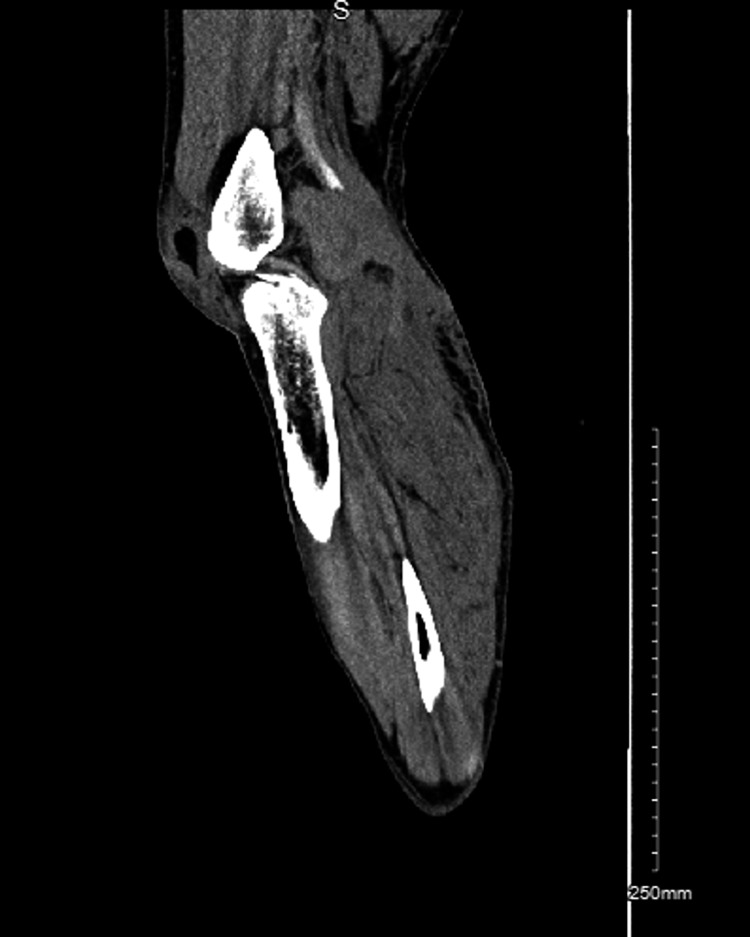
Sagittal view of CT angiography of the left leg showing a large hematoma in the popliteal fossa and abrupt non-opacification of the distal popliteal artery

The primary and secondary surveys were notable for moderate tenderness at the popliteal fossa, reduced range of motion at the knee, and diminished distal pulses (no hard signs of vascular injury or lacerations were noted). X-rays performed in the trauma bay showed lateral patella dislocation and a Type 5 Schatzker tibial plateau non-displaced fracture (Figure 1). A knee immobilizer was applied. The patient had no other injuries and remained hemodynamically stable. He was taken for a CT angiography of the LLE given the suspicion of vascular injury (as opposed to performing an ankle-brachial index (ABI) due to the mechanism of injury, worsening pain, and diminished pulses. If a normal ABI was obtained, it would not have precluded obtaining a CT angiogram, given our suspicion). The CT angiography of the LLE showed an abrupt non-opacification of the distal portion of the popliteal artery as it bifurcated into the anterior tibial and tibial-peroneal trunks with an overlying large hematoma in the left popliteal fossa (Figure 2). There were also comminuted fractures of the left femoral head (not reported on the X-rays obtained in the trauma bay).

**Figure 3:**
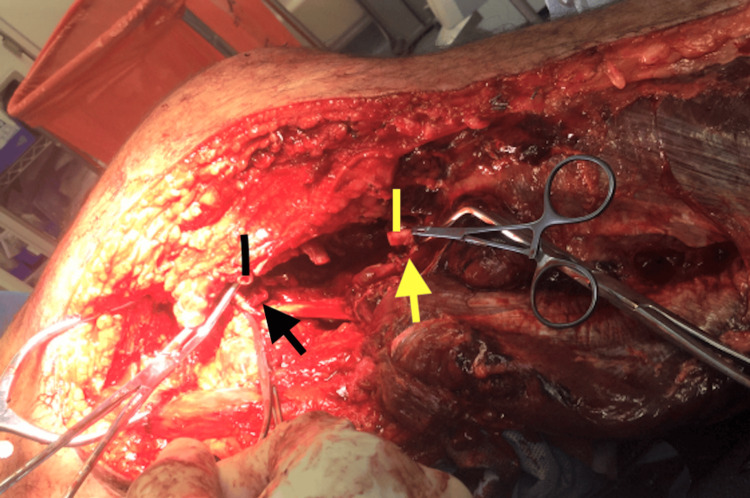
Intraoperative view of the popliteal artery (rectangle) and vein (arrow) transection with long defect. Proximal vessels are highlighted by the yellow rectangle and arrow while distal vessels are highlighted by the black rectangle and arrow.

The patient was then taken emergently to the operating room for exploration of the left popliteal fossa. The patient was positioned supine, with a plan to proceed with the medial approach to exposing the popliteal vessels. During the exploration, it was discovered that the patient also had a gastrocnemius muscle tendon transection proximally and a complete transection of the popliteal artery and vein with an approximately 5 cm defect for both (Figure 3).

**Figure 4:**
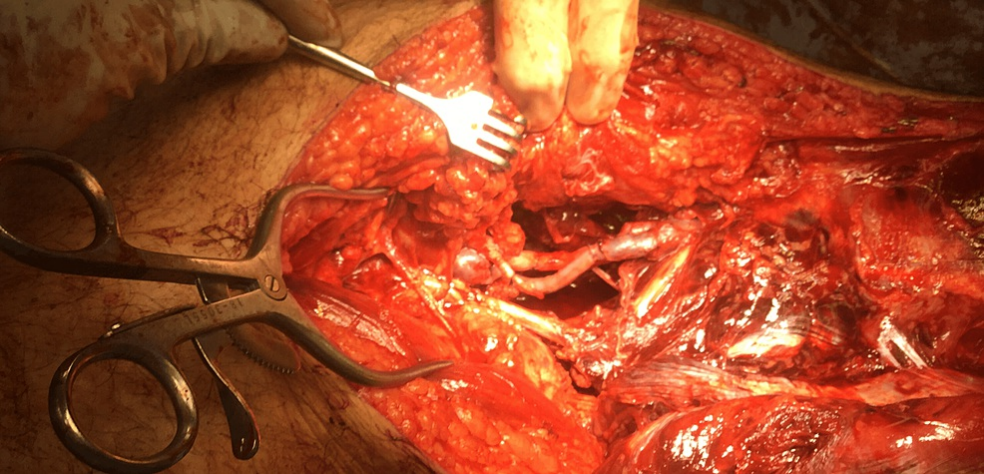
Intraoperative view of the repaired popliteal artery and vein with a reversed saphenous interposition graft

Given the large defect of both vessels, the decision was made to harvest approximately 12 cm of contralateral greater saphenous vein as a graft. The harvested greater saphenous vein was then used as a reversed interposition graft for both popliteal artery and vein repair. The end-to-end anastomoses were done using 6-0 polypropylene sutures, with enough length on the graft to allow tension-free anastomoses (Figure 4).

The gastrocnemius muscle was reattached to its insertion point. A four-compartment fasciotomy was performed on the left leg due to tense compartments (although <6 hours from injury), concurrent arterial/venous injury, and crush injury mechanism; the muscles appeared viable upon release of the compartments. The LLE dorsalis pedis pulse was palpable. Orthopedics then performed a knee-sparing external fixation and closed reduction of the patella dislocation. The patient's position did not need to be changed during the surgery. Postoperatively, the patient continued to have a palpable DP pulse, the leg was warm and well-perfused.

The patient underwent close monitoring in the surgical ICU, including frequent pulse checks. The patient continued to have a well-perfused LLE with palpable 2+ dorsalis pedis. The lateral fasciotomy wound was primarily closed, and the medial wound was managed with negative pressure wound therapy. The patient underwent a definitive fixation of the bicondylar tibial plateau fracture within two weeks. During re-evaluation of the femoral head fracture, it was not noted on repeat CT imaging and may have been an artifact reported on the CT angiogram. The patient was eventually discharged to a rehabilitation center.

## Discussion

Accounting for all the major traumas in urban trauma centers, extremity vascular injuries account for approximately 5% [1]. Most specifically, blunt trauma to the lower extremity is often associated with a 28-46% chance of injury to the popliteal artery. The types of injuries that usually occur are intimal, occlusion, or transection injuries [2]. These injuries occur due to traction or avulsion of the vessel, or directly due to bony fragments [3]. Mullenix analyzed the National Trauma Data Bank of 1,130,000 patients and identified a total of 1,395 PVI, which correlated to an incidence of 0.2% [4].

The popliteal artery and vein are positioned anatomically between the medial and lateral heads of the gastrocnemius and popliteus muscles. The popliteal fossa is bounded by the knee joint capsule and the popliteal surface of the femur as the floor. Therefore, in this location, the popliteal artery is most vulnerable to injuries, primarily due to fractures or joint dislocations [2,5].

PVI is associated with a significantly high risk for the development of compartment syndrome. Patients with prolonged ischemia time >6 hours, concomitant arterial and venous injuries, and combined vascular and skeletal injuries are at increased risk of compartment syndrome [7].

The diagnosis of lower extremity vascular injury is often and reliably made by physical examination. Most cases will present with clear signs of vascular injury, that is, “hard” signs [3]. These hard signs include active hemorrhage, absent distal pulses, distal ischemia, Bruit or thrill, and expanding or pulsatile hematoma.

Extremity pulse examination had a 100% sensitivity in predicting the presence of an arterial injury that required repair. A normal pulse examination had a 100% negative predictive value with an accuracy of 93.9 % [2]. However, any abnormal pulse detected on examination would require further evaluation with angiography or surgical exploration [2]. The presence of hard signs during the initial physical exam is a reliable indicator for immediate surgical exploration. Once there are hard signs, obtaining any vascular imaging is unnecessary and only delays definitive repair, contributing to prolonged ischemia and amputation rates [7]. Once a PVI has been identified, urgent surgical exploration is warranted.

The patient in our case report had a high-impact blunt trauma to the affected extremity without any knee dislocation or displaced fractures. The patient, however, had a non-expanding hematoma in the popliteal fossa and diminished distal pulse that warranted further examination with CT angiography (ABI was deferred due to a suspicion of vascular injury). Once the PVI was identified, prompt surgical exploration was carried out.

During surgical exploration for PVI, the patient should be prepped and draped, including both lower extremities. This is to aid in the harvesting of saphenous vein grafts from the contralateral extremity if needed [7].

The popliteal artery has three segments, which are the suprageniculate (above the knee), mid-popliteal (behind the knee), and infrageniculate (below the knee) segments. Exposure to each segment of the popliteal artery is distinct. Medial exposure of the supra geniculate segment begins with a longitudinal incision along the groove between the vastus medialis and sartorius muscles, which is about 1 cm posterior to the femur. Medial exposure of the infrageniculate segment is via a longitudinal incision 1 cm posterior to the tibia from the medial tibial condyle for the proximal third of the leg. The mid-popliteal segment can be exposed via a posterior (the patient is in a prone position) or medial approach, but in the setting of trauma, the medial approach is the most practical [8].

The vessel is then accessed through meticulous sharp and blunt dissection. Any devitalized segments of the vessels must be debrided to viable appearing vessels. An important step is to examine the intima of the artery for any intimal flaps [7]. The decision regarding primary arteriorraphy, primary end-to-end anastomosis, or placement of an interposition graft is based on the size of the injury (<30% of vessel circumference) or the length of a vessel lost (>2 cm) [3,4]. The incidence of long-segment vessel loss from blunt trauma has not been reported during our literature search, but it has been noted that blunt trauma is associated with a high risk of soft tissue loss [3].

Small lacerations of the artery can be repaired primarily. However, in the majority of cases, reconstruction with a reversed saphenous vein interposition graft is necessary [4]. The preferred suture for any repair or anastomosis of the popliteal artery is a 5-0 or 6-0 monofilament non-absorbable suture (polypropylene) in a running or interrupted fashion. Prior to any repair, Fogarty embolectomy catheters should be passed distally and proximally to ensure good backward and forward bleeding by removing all thrombus. Heparinized saline should also be irrigated into the proximal and distal ends to help prevent further thrombus formation [3,4]. Authors also recommend that completion angiography be performed if the dorsalis pedis and posterior tibial arteries are not palpable after vascular repair or reconstruction [4].

In some cases, a temporary intravascular shunt may be required for damage control if the patient is hemodynamically unstable and there are other life-threatening injuries requiring repair. This enables continuous venous outflow and arterial inflow to be maintained in the extremity during damage control surgery [3].

## Conclusions

In conclusion, we have reported a rare occurrence of concomitant popliteal artery and vein transection due to blunt lower extremity trauma. Literature has shown that although PVI incidence remains low, blunt PVI is associated with increased morbidity as compared to penetrating lower extremity trauma. A high index of suspicion therefore should be maintained for vascular injuries in all patients with blunt trauma to the lower extremities, with a systematic approach followed for appropriate workup and intervention. Minimizing delays in presentation to the trauma center, diagnosis, and revascularization are essential for limb salvage.
